# Longitudinal Predictors of Adaptive Functioning in Emerging Adults with and without Autism Spectrum Disorder

**DOI:** 10.1007/s10802-024-01265-y

**Published:** 2024-11-15

**Authors:** Ingrid Nesdal Fossum, Merete Glenne Øie, Stian Orm, Per Normann Andersen, Erik Winther Skogli

**Affiliations:** 1 Division Mental Health Care, Innlandet Hospital Trust, Lillehammer, Norway; 2 Department of Psychology, University of Oslo, Oslo, Norway; 3 Research Department, Innlandet Hospital Trust, Brumunddal, Norway; 4 Department of Psychology, Inland Norway University of Applied Sciences, Lillehammer, Norway

**Keywords:** Autism spectrum disorder, Executive functions, Adaptive functioning, WFIRS, Longitudinal study

## Abstract

Individuals with autism spectrum disorder (ASD) display heterogeneity in adaptive functioning, underscoring the need to identify predictors to inform clinical and scientific interventions. We investigated the longitudinal associations between an autism diagnosis, co-occurring psychopathology symptoms, executive functions (EF) and subsequent adaptive functioning in individuals with and without ASD (IQ > 70). Sixty-six individuals (26 with ASD, 40 without ASD) were assessed at baseline (mean age = 11.8 years, SD = 2.1) and at 10-year follow-up (mean age 21.4, SD = 2.3). The diagnostic evaluation comprised a comprehensive assessment of autism symptoms and emotional and cognitive functioning. Co-occurring psychopathology symptoms were assessed with two measures: self-reported depressive symptoms with the Short Mood and Feelings Questionnaire and parent-reported total problems with the Child Behavior Checklist 6–18. Participants completed neuropsychological tests to evaluate EF. We investigated adaptive functioning by using the Weiss Functional Impairment Rating Scale (WFIRS) which is a self-report measure of impairment in the following domains: family, work, school, life skills, self-concept, social and risk-taking. Among the emerging adults previously diagnosed with ASD, 46% reported living independently, 75% had at least one friend, and 71% were employed or in education. Individuals with ASD reported significantly lower adaptive functioning compared to individuals without ASD (WFIRS Total, Hedges’ g = 0.92). Greater EF difficulties in childhood/adolescence predicted lower adaptive functioning in emerging adulthood, surpassing the influence of autism diagnosis and co-occurring symptoms. The findings highlight the influential role of EF, implying that interventions targeting EF difficulties could improve long-term outcomes for individuals with ASD.

Autism spectrum disorder (ASD) is a complex condition characterized by genetic and phenotypic heterogeneity, resulting in variable degrees of severity, symptomatology, and outcomes. For many individuals who are diagnosed with ASD^1^, it is a lifelong and severe condition that impacts the individual, their families and society, leading to functional difficulties in various domains, including school, work, and family life throughout their lifespan (Howlin & Magiati, 2017; Lai et al., 2014). Adaptive functioning refers to conceptual, practical and social skills that have been learned and are performed to meet the demands of everyday living (APA, 2013). Research suggests that there may be a widening gap in adaptive functioning between children and adolescents with ASD and typically developing (TD) individuals as they face increased environmental demands with age (Pugliese et al., 2015). While there may be reduction in autism symptoms over time (Hartman et al., 2016; Helles et al., 2015; Orm et al., 2022b*), the adaptive functioning associated with ASD can still present challenges.^2^ Therefore, it is essential to examine the adaptive functioning of individuals with ASD in emerging adulthood to better understand the overall outcomes of ASD (Lau-Zhu et al., 2019). Emerging adulthood is a developmental stage where individuals gradually gain autonomy and assume the responsibilities of adulthood (Arnett, 2000).

Reviews have pointed at great variability in adaptive outcomes among adults who have been diagnosed with ASD, with nearly 50% (with or without intellectual disability (ID) experiencing good or fair outcomes in terms of independent living, friendship, education, and occupation (Howlin & Magiati, 2017; Magiati et al., 2014; Mason et al., 2021; Steinhausen et al., 2016). Longitudinal studies following individuals with an ASD diagnosis without ID over time indicate that 41–82% show a good or fair outcome in adult age (Cederlund et al., 2008; Farley et al., 2009; Howlin et al., 2013). In a 20-year follow-up study of individuals with ASD without ID, 27% reported having a full-time job, and 12% reported living independently at average age 32 years (Farley et al., 2009). In another longitudinal study of males with ASD, rates of independent living increased markedly from the average age of 21 to around 30 years (from 37 to 62%; Cederlund et al., 2008; Helles et al., 2017). Moreover, 40% were either employed or enrolled as a student, 48% had two or more friends, and 30% had a partner (Helles et al., 2017). Research from Denmark has shown that the rates of completing high school is substantially lower among individuals with ASD compared to individuals without ASD (35% vs. 78%; Toft et al., 2021). Aydin and colleagues found that ASD was associated with overall functional impairment in an adult twin sample with a mean age of 22 years (Aydin et al., 2022). For subdomains of adaptive functioning, ASD was associated with more impaired social function, and with lower risk-taking (Aydin et al., 2022).

Knowledge about factors influencing adaptive functioning is crucial for understanding the long-term impact of ASD, serving both research and clinical purposes. Among relevant factors at the individual level are the ASD diagnosis and the symptoms in themselves. In a systematic review on individuals with ASD, the ASD diagnosis and autism symptom severity were identified as significant longitudinal predictors of later negative outcomes (Kirby et al., 2016). Notably, the reduction of autism symptoms is not always followed by improvement in adaptive functioning in adult age (Copeland et al., 2015). This could indicate that factors apart from autism symptoms and diagnosis are of importance for adaptive functioning for individuals who are diagnosed with ASD. Indeed, a recent study found that the relationship between an ASD diagnosis in childhood and reduced quality of life in emerging adulthood was fully mediated by internalizing difficulties in adolescence (Andersen et al., 2023*). Thus, one potential factor related to lower adaptive functioning among individuals with ASD is the presence of co-occurring psychopathology symptoms.

Co-occurring psychopathology constitutes a central source of the problems experienced by individuals with ASD, with more than 70% of children and adolescents showing co-occurring symptoms (Lai et al., 2014). Recent findings from a systematic review and meta-analysis found pooled prevalence estimates of 18% for depressive disorders in individuals diagnosed with ASD (Micai et al., 2024), alongside high prevalences for psychopathology symptoms generally (Lai et al., 2014; Micai et al., 2024). Empirical evidence indicates a significantly reduced level of function when comorbid psychiatric disorders are present in individuals with ASD (Mattila et al., 2010), it has even been suggested that co-occurring psychiatric disorders have a greater impact on prognosis and function than ASD symptoms do (Gillberg & Fernell, 2014). Research is scarce on the potential impact of co-occurring psychopathology on later adaptive functioning in individuals diagnosed with ASD. One longitudinal study of children diagnosed with ASD found no association between emotional and behavioral problems at age 7 years (mean) and adaptive functioning at age 13 (Chandler et al., 2022). A cross-sectional study on adults with ASD without ID found a link between lower adaptive functioning and symptoms of depression, anxiety and ADHD, and proposed that this should be further explored in longitudinal studies (Kraper et al., 2017). Interestingly, co-occurring psychopathology has been identified as a longitudinal predictor of functional impairment in individuals with other neurodevelopmental disorders such as ADHD (Orm et al., 2023*).

Co-occurring psychopathology symptoms among individuals with ASD are unstable and wane and wax over time (McCauley et al., 2020), and likewise, autism symptom presentation may fluctuate over time (Magiati et al., 2014). In contrast, difficulties with executive functions (EF) have been identified as a promising endophenotype in ASD and as a relatively stable, underlying vulnerability (Craig et al., 2016; Hartman et al., 2016). Difficulties with EF have been reported in children, adolescents, and adults with ASD (Craig et al., 2016; Demetriou et al., 2017). EF are commonly referred to as a set of higher order cognitive abilities necessary for goal-directed behavior, linked to prefrontal and temporal brain regions (Miyake et al., 2000). EF difficulties are associated with poor behavioral, social, educational, and occupational outcomes in ASD populations (O’Hearn et al., 2008). Longitudinal studies have shown that EF difficulties tend to persist among individuals with ASD, despite maturation across time, and despite a decline in autism symptomatology (Fossum et al., 2021*; Hartman et al., 2016). Thus, empirical evidence indicates that EF difficulties are mainly trait-dependent rather than state-dependent phenotypes (Hartman et al., 2016). Individual differences in EF may be one potential source of the heterogeneity in adaptive functioning outcomes for individuals diagnosed with ASD (Pellicano, 2012). While there have been studies linking EF to concurrent adaptive function in individuals with ASD (Nyrenius & Billstedt, 2020; Pugliese et al., 2016; Wong et al., 2023), longitudinal studies on this topic are rare. One study, however, found that better EF in childhood predicted better adaptive behavior 12 years later in individuals with ASD without ID (Kenny et al., 2019). This led the authors to propose EF as a potential prognostic indicator among individuals with ASD (Kenny et al., 2019).

## Knowledge Gap

Gaining a thorough understanding of the unique developmental phase of emerging adulthood is crucial for evaluating outcomes for individuals diagnosed with ASD (Lau-Zhu et al., 2019). Emerging adulthood is characterized by expectations of increased autonomy and responsibility, and reduced external support (Arnett, 2000; Turgay et al., 2012). Given the developmental challenges posed by ASD, including a higher mortality risk compared to individuals without ASD, identifying predictors of later adaptive functioning is essential (Hartman et al., 2016; Steinhausen et al., 2016; Woolfenden et al., 2012). Most existing studies have focused on individuals with ASD and ID or borderline intellectual functioning, or individuals diagnosed in preschool years (for instance Bal et al., 2015; Clarke et al., 2021; Forbes et al., 2023; Szatmari et al., 2015; Pickles et al., 2020). There is a need for research on longitudinal predictors of adaptive functioning for individuals with ASD without ID (Howlin & Magiati, 2017; Kirby et al., 2016; Steinhausen et al., 2016). Understanding these predictors may have important clinical implications related to treatment planning and implementation across the lifespan, as well as for providing prognostic information to parents and teachers post-diagnosis (Hartman et al., 2016).

## The Current Study

This study had two primary aims. First, we aimed to describe indicators of adaptive functioning among emerging adults who had been followed prospectively for 10 years after being diagnosed with ASD in childhood or adolescence. Indicators of adaptive functioning were assessed in terms of living situation, education and occupation, friendships, and partner relationships. Second, we investigated if autism diagnosis, co-occurring psychopathology symptoms, and EF could predict self-reported adaptive functioning in emerging adulthood. Autism diagnosis and co-occurring psychopathology symptoms in childhood were included because of their association with impaired functioning in individuals with ASD. EF were included because of their link to functioning across various domains and their potential role as a long-lasting cognitive vulnerability in individuals with ASD. Co-occurring psychopathology symptoms and EF have been proposed as influential factors for adult outcomes of individuals with ASD, warranting further investigation (Clarke et al., 2021). We hypothesized that an autism diagnosis, co-occurring psychopathology symptoms, and greater EF difficulties at baseline would predict lower adaptive functioning at the 10-year follow-up.

## Methods

The data used in this study was collected as part of the Lillehammer Neurodevelopmental Follow-Up Study (LINEUP), where diagnostic, functional, and neuropsychological assessments were conducted in three waves: baseline (T1) in 2009–2010, 2-year follow-up (T2) in 2011–2012, and 10-year follow-up (T3) in 2018–2020 (Andersen et al., 2013*; Fossum et al., 2021*). In the current study, we used data from the baseline and 10-year follow-up assessments for participants who took part at both assessments.

### Participants and Procedure

#### Baseline

Children and adolescents were recruited from Child and Adolescent Mental Health Outpatient Clinics in Norway, upon consecutive referrals with suspicion of a neurodevelopmental disorder. The final sample consisted of 38 individuals with an ASD diagnosis. Eight participants in the ASD group met the criteria for comorbid ADHD, of whom three used stimulant medication. The sample also included a comparison group of 50 typically developing (TD) children/adolescents, who were recruited from local schools. See Table 1 for demographic and clinical characteristics. Data on race/ethnicity was not collected.

**Table 1:** Demographic and clinical characteristics for the ASD and TD groups at baseline and 10-year follow-up

Baseline	ASD *n* = 26		TD *n* = 40		Group comparison	
	M	SD	M	SD	*t*	Hedges’ g
Sex (male/female)	21/5		26/14			
Age (years)	12.3	2.4	11.5	2.1	1.44	0.38
FSIQ	101.0	19.2	104.5	13.1	-0.83	-0.22
Mother’s education (years)	12.9	2.8	14.7	2.3	-2.89**	-0.72
Autism symptoms ^a^	23.3	9.1	1.5	1.6	12.05***	3.69
Depressive symptoms ^b^	6.8	5.9	2.4	2.3	3.70***	1.08
Total problems ^c^	65.3	9.8	37.5	8.9	11.8***	2.96
Executive functions ^d^	2.25	2.6	-0.1	2.6	3.49***	0.89
**10-year follow-up**						
Age (years)	22.2	2.6	20.9	1.9	2.15*	0.57
FSIQ	109.3	18.3	115.4	12.0	-1.44	-0.41

Note. ASD = autism spectrum disorder; TD = typically developing, FSIQ = Full-Scale Intelligence Quotient estimated from Wechsler Abbreviated Scale of Intelligence. ^a^ Measured with the Autism Spectrum Screening Questionnaire. ^b^ Measured with the Short Mood and Feelings Questionnaire. ^c^ Measured with the Child Behavior Checklist 6–18, *N* = 39 for TD group. ^d^ Composite score where higher scores indicate more difficulties, calculated from results on the Letter-Number Sequencing Test from Wechsler Intelligence Scale for Children- IV, the Color-Word Interference Test, condition 3, and the Trail Making Test, condition 4, both from Delis-Kaplan Executive Function System. * *p* <.05, ** *p* <.01, *** *p* <.001

All participants underwent diagnostic assessments based on separate interviews with children and parents, using the Schedule for Affective Disorders and Schizophrenia for School Aged Children/Present and Lifetime version-2009 (K-SADS-PL; Kaufman et al., 1997). Parents filled out the Autism Spectrum Screening Questionnaire (ASSQ; Ehlers et al., 1999), the ADHD Rating Scale–IV (ASR-IV; DuPaul et al., 1998), and the Child Behavior Checklist (CBCL; Achenbach & Rescorla, 2001). After evaluation of information from K-SADS-PL, self-reports, parent reports, and teacher reports on academic and social functioning, an ASD diagnosis was assigned if criteria from DSM-IV were met. See Andersen et al., 2013* for details on recruitment strategy and diagnostic assessment. The exclusion criteria for all participants were prematurity (< 36 weeks gestational age), Full Scale IQ (FSIQ) estimate below 70, and neurological disease. We used the Wechsler Abbreviated Scale of Intelligence (WASI; Wechsler, 1999) to estimate FSIQ. For the TD group, additional criteria were no history of psychiatric disorder, dyslexia, or head injury with loss of consciousness. The comprehensive clinical, diagnostic, and neuropsychological assessment was repeated at the 2-year follow-up. At T2, 15 (41%) in the ASD group were receiving special education services. Data from T2 are not included in the current paper.

#### 10-Year Follow-Up

The diagnostic and functional assessment was repeated after 10 years using age-appropriate instruments (Fossum et al., 2021*). From the original sample, 26 with ASD and 40 TD individuals took part at 10-year follow-up (retention rate 75%), and these 66 participants were included in the analysis in the current paper. From the eight participants at baseline who met diagnostic criteria for both ASD and ADHD, five participated at 10-year follow-up. Baseline differences between T3 participants and those who dropped out were investigated using independent samples t-test. The results showed that the groups did not differ on the baseline characteristics age, IQ, mothers’ educational level, or autism symptoms, but the T3 participants had better baseline EF than those who opted out (*p* =.024). Previous research from the LINEUP has reported that 48–78% of the individuals who were diagnosed with ASD in childhood/adolescence still met the diagnostic ASD criteria in emerging adulthood (Orm et al., 2022a). The TD participants were screened for symptoms of the most common psychiatric disorders using the MINI-screen interview (Sheehan et al., 1998) but were not re-screened for ASD symptoms at 10-year follow-up.

### Ethics

We conducted the study following the Helsinki Declaration of the World Medical Association Assembly. Participation was voluntary. At T1 and T2, children below 12 years of age gave verbal consent before inclusion, children aged 12 years gave informed written consent, and for all participants below 16 years of age, their parents gave informed written consent. At T3, all participants gave their informed written consent. The study was approved by the Regional Committee for Medical Research Ethics in Eastern Norway (T1 and T2: REK Øst-Norge 6-2009-24, T3: 2017/2036/REK Sør-Øst) and the privacy ombudsman for research at the Innlandet Hospital Trust (no. 95495).

### Measures

#### Co-Occurring Psychopathology Symptoms at Baseline

We used the Short Mood and Feelings Questionnaire (SMFQ), which is a 13-item self-report instrument designed to measure depressive symptoms in children 8 to 18 years of age (Angold et al., 1995). The participants respond on a three-point scale (“not true”, “sometimes true” and “true”). We used the total score, where higher raw scores indicate more depressive symptoms. The SMFQ has demonstrated good psychometric properties with high internal consistency and inter-item reliability, and test-retest stability (Cronbach’s alpha ≥ 0.89; Costello & Angold, 1988; Costello et al., 1991; Fjermestad et al., 2020; Kuo et al., 2005). Regarding psychometric properties for the SMFQ for ASD populations specifically, there are reports of good internal consistency at α = 0.85 among adolescents with ASD, similar to the internal consistency for TD controls (Mazefsky et al., 2014).

We used an instrument from the Achenbach System of Empirically Based Assessment (ASEBA) for assessing emotional and behavioral problems (Achenbach & Rescorla, 2001). Parents filled out the Child Behavior Checklist 6–18 (CBCL). CBCL consists of 113 items which are scored on a 3-point scale (0 = absent, 1 = occurs sometimes, 2 = occurs often). We used the total problems score which is computed from the two broadband scores internalizing and externalizing symptoms. We used the total problems T-score (M = 50, SD = 10) based on American norms, where higher scores indicate more symptoms. The Norwegian version of CBCL has good levels of reliability (≥ 0.80), sensitivity (40–83%), specificity (70–94%) (Kornør & Jozefiak, 2012). There is empirical support for the factor structure, the reliability of scales and subscales (λ–2 = 0.90) and good sensitivity (0.91) in identifying emotional problems in youth with ASD (Pandolfi et al., 2012).

#### Executive Functions at Baseline

We computed a global composite score of EF from the participants’ results on three neuropsychological tests assumed to assess core EF components (Bagetta & Alexander, 2016; Miyake et al., 2000). The Letter/Number Sequencing Test from the Wechsler Intelligence Scales for Children-IV (WISC-IV; Wechsler, 2004) was used to measure working memory. The Color-Word Interference Test, Condition 3, from the Delis Kaplan Executive Function System (D-KEFS; Delis et al., 2001) was used to measure inhibition. The Trail Making Test, Condition 4, from D-KEFS (Delis et al., 2001) was used to measure flexibility. We used the procedure described by Orm et al. (2022b) to compute the EF composite measure. For each test separately, raw scores were converted to Z scores based on the baseline mean and standard deviation in the TD group. The new variables correlated perfectly with the original variables and retained all inter-individual variance. Higher Z scores indicate more EF difficulties. The three Z scores were summed to create the EF composite score; thus, each test exerts the same amount of influence on the composite measure. Using a composite measure instead of separate tests reduced the number of variables and thus increased statistical power (Øie et al., 2021; Romer & Pizzagalli, 2021). There is also greatest empirical support for a unidimensional model of EF in children and adolescents (Karr et al., 2018).

#### Adaptive Functioning at 10-Year Follow-Up

In this study we operationalized adaptive functioning by using a measure of functional impairment. Specifically, we assessed functional impairment through self-reports obtained via the Weiss Functional Impairment Rating Scale (WFIRS; Weiss et al., 2005). This questionnaire consists of 69 items collecting the participants’ perspectives on their functioning in seven domains: Family relations (8 items), Work adjustment (11 items), School functioning (10 items), Life Skills (12 items), Self-Concept (5 items), Social functioning (9 items) and Risk-taking (14 items). The participants rated each item on the extent to which emotional or behavioral problems have impacted functioning during the last month on a Likert scale with alternatives “not at all or never” = 0, “sometimes or to some degree” = 1, “often or a lot” = 2, “very much or very often” = 3, with a final alternative “not applicable”. We used the total functional impairment score based on the seven subscales, with higher scores indicating more functional impairment. In samples of individuals with ADHD and the general population, the WFIRS shows strong psychometric qualities of reliability (α = 0.79–0.96), factor structure, and convergent validity (e.g., Canu et al., 2020; Haugan et al., 2021). The WFIRS has not yet been validated in an ASD population, but calculations from the current ASD sample showed acceptable to excellent internal consistency for six out of seven domains (α > 0.71), and excellent internal consistency for the total scale (α = 0.90), while the risk domain showed poor internal consistency (α = 0.56).

#### Indicators of Adaptive Functioning

Moreover, we assessed indicators of adaptive functioning using selected items from the Adult Self-Report scale (ASR; Achenbach & Rescorla, 2003) concerning education and occupation, friendships, and partner relationships. Educational level was assessed by asking participants to report their completed studies from a list of 10 categories (including one open-endedcategory), where we dichotomized the responses into a yes/no variable indicating whether they had completed high school. For targeting current educational/occupational status, we combined responses from questions asking if the participants had been in education in the last six months (yes/no) and if they had had paid work in the last six months (yes/no). This yielded a variable of having been in education or paid work in the last six months (yes/no). Friendships were assessed by asking how many close friends they had (excluding family members), where responses on four categorized were dichotomized into a variable having at least one close friend (yes/no). Partner relationships were assessed by asking participants to specify their marital status, with responses categorized into six separate options (including one open-ended). These were dichotomized into a variable indicating whether they were in a partner relationship (yes/no). In addition, we asked participants about their living situation using self-made questions. One question inquired about their type of housing over the last three months (private housing, public housing, shelter, supervised housing, or institution), and another asked about their living arrangement over the last six months (spouse/cohabiting partner, single parent with child, with parents/relatives, alone, or institution). These responses were combined into a variable where living independently (yes/no) was defined as living in private housing alone (with or without children) or with spouse/cohabiting partner.

### Statistical Analyses

All data analyses were performed in SPSS version 29 for Windows. We investigated demographic characteristics at baseline and 10-year follow-up, using the Chi-square test for independence and t-tests comparing the ASD and the TD group. The baseline sample was restricted to participants who also took part at 10-year follow-up (*N* = 66). Significant results are reported at *p ≤*.05 level. To describe the adaptive functioning in the ASD group in more detail, we report data on living situation, education and occupation, friendships, and partner relationships. Data on education and occupation, friendships and partner relationships were compared between the ASD and TD group using Chi-square tests.

We compared the self-reported adaptive functioning scores (WFIRS total and subdomain scores) across the groups (ASD vs. TD) using independent samples t-tests. We used Hedges’ g to measure effect size, interpreting a Hedges’ g of 0.20, 0.50, and 0.80 as a small, medium, and large effect size, respectively (Cohen, 1992). A positive Hedges’ g indicates lower adaptive functioning in individuals with ASD relative to the TD comparison group.

We ran a Pearson bivariate correlation analysis to determine the associations between depressive symptoms (T1), total problems (T1), EF composite score (T1), and functional impairment (WFIRS, T3). Pearson’s *r* of 0.10, 0.30, and 0.50 can be interpreted as a small, medium, and large effect size (Cohen, 1992). We computed a composite score for EF using three neuropsychological tests. To do so, we converted raw scores to Z scores based on the baseline (T1) mean and standard deviation from the TD group. This method ensured that the new variables correlated perfectly with the original variables and retained all inter-individual variance (Orm et al., 2022*). Higher scores indicate more EF difficulties. For each participant, the three Z scores were summed, to create an overall EF composite score. The three tests displayed medium to high correlations (*r* =.43 −.63, *p* <.01). The conversion procedure was necessary because the three separate EF tests use different scales and yield result in opposite directions. This method also ensured that each test had the same influence on the composite score.

Further, we ran two separate multiple regression analyses to examine the joint and independent contribution of autism diagnosis, co-occurring psychopathology symptoms and EF composite score to the prediction of total functional impairment (WFIRS). In the first model, we used the self-reported depressive symptoms measure (SMFQ), while in the second, we used the parent-reported total problems measure (CBCL). Age and mother’s education were included as covariates in each model. The models also included two interaction terms between group (ASD = 1, TD = 0) and psychopathology symptoms (SMFQ or CBCL), and group and EF, to examine if the effects of psychopathology or EF varied by group (ASD or TD). We ran the regression analyses for the entire sample (ASD and TD) because this yielded a higher N and more variance in the predictor and outcome variables, thus contributing to higher statistical power (Agresti & Finlay, 2009). Pairwise deletion was chosen to address missing data.

## Results

Table 1 presents the demographic characteristics for the ASD and TD groups at baseline and 10-year follow-up, with comparison. Table 2 presents indicators for adaptive functioning for the ASD and TD group at 10-year follow-up. Among the individuals in the ASD sample, 46% reported living independently, 75% had completed high school, 71% were either employed or currently enrolled as a student, 17% reported having a partner, and 75% had at least one friend. Chi-square tests showed that a higher proportion reported having at least one close friend in the TD group (100% vs. 75%, *p* <.001). Moreover, there was a higher proportion in the TD group that reported having either a paid job or being in education 98% vs. 71%, *p* =.002). There were no statistically significant differences in the proportion that reported having a partner relationship, nor for having completed high school.

**Table 2:** Indicators of adaptive functioning for the ASD and TD groups at 10-year follow-up

	ASD	TD	χ2	*p*
Living independently	11 (45.8%)			
Having at least one close friend ^a^	18 (75.0%)	40 (100%)	11.03	< 0.001
Having a partner relationship ^a^	4 (16.6%)	8 (20%)	0.11	0.741
Completed high school ^a^	18 (75.0%)	35 (89.7%)	2.41	0.120
Having a paid job or in education ^a^	17 (70.8%)	39 (97.5%)	9.75	0.002

Note. ASD = autism spectrum disorder. TD = typically developing. Living independently defined as living in private housing alone or with a partner, *n* = 26. ^a^ From adult self-report (ASR), *n* = 24

### Adaptive Functioning in Emerging Adults with and Without ASD

The results of comparing adaptive functioning total and subdomain scores across groups are shown in Table 3. The ASD group reported significantly lower adaptive functioning (higher WFIRS total score) than the TD group (Hedges’ *g* = 1.03). The ASD group also reported significantly lower adaptive functioning in the domains of family relations, work adjustment, life skills, self-concept, and social functioning compared to the TD group, with medium to large effect sizes (Hedges’ *g* = 0.71–1.29).

**Table 3:** Adaptive functioning at 10-year follow-up across ASD and TD groups

Variable	ASD *N* = 26	TD *N* = 40	Group comparison	
	M (SD)	M (SD)	*t*	Hedges’ g
**WFIRS Total**	0.60 (0.34)	0.31 (0.22)	3.75***	1.03
WFIRS Family	0.59 (0.44)	0.26 (0.24)	3.40**	1.00
WFIRS Work	0.43 (0.34)	0.23 (0.24)	2.39*	0.71
WFIRS School	0.58 (0.45)	0.38 (0.36)	1.64	0.49
WFIRS Life Skills	0.75 (0.40)	0.43 (0.30)	3.73***	0.93
WFIRS Self-concept	1.13 (0.87)	0.53 (0.54)	3.16**	0.87
WFIRS Social	0.71 (0.52)	0.22 (0.23)	4.50***	1.29
WFIRS Risk	0.16 (0.17)	0.23 (0.24)	-1.20	-0.30

Note. ASD = autism spectrum disorder, TD = typically developing, WFIRS = Weiss Functional Impairment Rating Scale, higher raw scores indicate more impairment.

*p <.05, ** *p* <.01, *** *p* <.001

### Longitudinal Predictors of Adaptive Functioning

Zero-order correlations between study variables group (ASD = 1, TD = 0), SMFQ, CBCL total problems, EF composite, and WFIRS total ranged from 0.33 − 0.83, all significant at *p* <.01. Results from the first regression analysis with baseline age, mother’s education, autism diagnosis, self-reported depressive symptoms, EF, group x depressive symptoms, and group x EF as predictors and functional impairment as outcome variable are shown in Table 4. All these variables together explained a significant and large amount of variance in total functional impairment at the 10-year follow-up (*F* (7, 56) = 4.26, *p* <.001, *R*^2^ = 0.35, *f*^2^ = 0.53). More EF difficulties were a significant predictor of lower adaptive functioning (β = 0.42, *t* = 2.09 *p* =.042). The interaction terms were non-significant, thus there was no indication that the predictive impact of EF on adaptive functioning varied by group.

**Table 4:** Baseline autism diagnosis, self-reported depressive symptoms, and EF as predictors of adaptive functioning (WFIRS) at the 10-year follow-up

Predictors from T1		B	95% CI	SE
Age in years		0.04*	[0.00, 0.07]	0.02
Mother’s education in years		− 0.00	[-0.03, 0.02]	0.01
Group (ASD = 1, TD = 0)		0.17	[-0.06, 0.39]	0.11
Depressive symptoms		0.03	[-0.02, 0.06]	0.02
Executive functions		0.04*	[0.00, 0.08]	0.02
Group x SMFQ		− 0.01	[-0.05, 0.04]	0.02
Group x Executive Functions		− 0.02	[-0.07, 0.03]	0.03

*Note.* Dependent variable = Weiss Functional Impairment Rating Scale Total score. Depressive symptoms measured with the Short Mood and Feelings Questionnaire, self-report. Executive Functions = Composite score calculated from results on the Letter-Number Sequencing Test from Wechsler Intelligence Scale for Children- IV, the Color-Word Interference Test, condition 3, and the Trail Making Test, condition 4, both from Delis-Kaplan Executive Function System. *N* = 66

*p <.05, ** *p* <.01, *** *p* <.001

Results from the second regression analysis with baseline age, mother’s education, autism diagnosis, parent-reported total problems, EF, group x total problems, and group x EF as predictors, are shown in Table 5. All these variables together explained a significant and large amount of variance in total functional impairment at the 10-year follow-up (*F* (7, 56) = 4.04, *p* <.001, *R*^2^ = 0.34, *f*^2^ = 0.51). More EF difficulties were a significant predictor of lower adaptive functioning (*β* = 0.42, t = 2.07, *p* =.043). The interaction terms were non-significant (*p* >.110), thus there was no indication that the predictive impact of EF on adaptive functioning varied by group in this model either.

**Table 5:** Baseline autism diagnosis, parent-reported total problems, and EF as predictors of functional impairment (WFIRS) at the 10-year follow-up

Predictors from T1		B	95% CI	SE
Age in years		0.04*	[0.01, 0.08]	0.02
Mother’s education in years		− 0.01	[-0.04, 0.02]	0.01
Group (ASD = 1, TD = 0)		0.83	[-0.01, 1.67]	0.42
Total problems		0.01	[-0.00, 0.02]	0.01
Executive functions		0.04*	[0.00, 0.08]	0.02
Group x Total problems		− 0.01	[-0.03, 0.00]	0.01
Group x Executive Functions		− 0.02	[-0.07, 0.03]	0.03

*Note.* Dependent variable = Weiss Functional Impairment Rating Scale Total score. Total problems from the Child Behavior Checklist 6–18, parent-report. Executive Functions = Composite score calculated from results on the Letter-Number Sequencing Test from Wechsler Intelligence Scale for Children- IV, the Color-Word Interference Test, condition 3, and the Trail Making Test, condition 4, both from Delis-Kaplan Executive Function System. *N* = 66

*p <.05, ** *p* <.01, *** *p* <.001

## Discussion

The current study contributes with important knowledge about adaptive functioning in adults who were diagnosed with ASD as children or adolescents. A positive finding was that about three-fourths in the ASD sample had completed high school, were currently studying or employed, and had at least one friend, while about half of the sample lived independently. Our findings are in line with reports that nearly 50% of adults with ASD have a fair or good outcome in terms of independent living, occupation, and friendship (Howlin & Magiati, 2017; Steinhausen et al., 2016). A notably higher proportion in the current sample reported living independently than in a 20-year longitudinal study of adults with ASD without ID (46 vs. 12%; Farley et al., 2009). In another longitudinal study of males with Aspergers syndrome, 37% were living independently at the mean age of 21.5 years (Cederlund et al., 2008), increasing to 62% living independently about 8 years later (Helles et al., 2017). The opportunity of independent living is closely related to educational, occupational, and financial situation, and the Norwegian context is unique in the sense that student loans, scholarships and no tuition fees allow for independent living while being a student. We should also note that proportions are not directly comparable across studies due to different samples and measures.

In our ASD sample, 75% had successfully completed high school (13 years of education), which is comparable to recent statistics from Norway where 81% of the general population completes high school (Statistics Norway, 2024). This finding contrasts with results of a Danish registry study which reported that 52% of individuals with Aspergers syndrome had completed high school by the age of 25 compared to 78% completion among individuals without ASD (Toft et al., 2021). Our data is however not detailed enough that we can tell whether the participants had achieved all learning objectives in the curriculum (thus received standard diploma). Approximately 70% of the individuals in the current ASD sample were either employed, enrolled in educational programs, or engaged in a combination of work and studies. This proportion is notably higher than the 40% reported in a follow-up study of males with ASD at an average age of 30 years (Helles et al., 2017). Farley et al. (2009) reported that 27% had full-time jobs at an average age of 32. In terms of social outcomes within our sample, although clearly lower than the TD group, a substantial majority of the individuals in the ASD group (75%) reported having at least one close friend. A notable proportion of the ASD group (17%), and similar to the TD group, reported being in partner relationship. In comparison, concerning Swedish males with ASD at an average age of 30 years, 48% reported having two or more friends, and 30% reported having a partner, according to research by Helles et al. (2017). Again, direct comparison should be avoided but together these findings contribute to illuminate adult outcomes for individuals diagnosed with ASD in childhood or adolescence.

One relevant factor for understanding the positive outcomes observed in the current sample could be the age at which the individuals with ASD were diagnosed. The sample in our study was diagnosed at a relatively old age, while earlier diagnosis tends to correlate with indicators of more atypical early development, higher levels of autism symptoms, and greater functional difficulties in childhood (Daniels et al., 2014; Mandell et al., 2005). Future studies could explore the potential impact of environmental factors such as differences in health care services and educational systems across locations and over time on the outcomes in this population. Investigating these variables could provide valuable insights into whether they contribute to the observed variation between different samples and geographic regions.

### Lower Adaptive Functioning in Emerging Adults with ASD

The individuals in the ASD group reported significantly lower adaptive functioning compared to the TD group, applying to subdomains of family relations, work adjustment, life skills, self-concept and social function, with medium to large effect sizes. Our results align with previous empirical findings suggesting that individuals with ASD face challenges in various aspects of life in adulthood (Farley et al., 2009; Howlin & Magiati, 2017; Lai et al., 2014; Steinhausen et al., 2016). Social impairment is a criterion for receiving the ASD diagnosis, and the findings suggest that more social challenges were still present 10 years after receiving the diagnosis compared to the TD group (American Psychiatric Association, 2013; Lai et al., 2014).

Previous research from the LINEUP reported a reduction in autism symptoms in the current ASD sample by emerging adulthood, where a subset of individuals no longer met criteria for ASD in emerging adulthood (Orm et al., 2022a*). The current study findings are in line with previous reports indicating persistent difficulties with adaptive functioning despite a decrease in symptoms among individuals diagnosed with ASD relative to TD individuals (Copeland et al., 2015; Hartman et al., 2016).

### Longitudinal Predictors of Adaptive Functioning

In this study, more EF difficulties stood out as a significant predictor of later lower adaptive functioning, a finding that supports and extends the existing research and the notion of early EF as a prognostic indicator (Kenny et al., 2019; Pellicano, 2012, 2013). Relatedly, a link between behavior-ratings of EF and adaptive functioning has been demonstrated in cross-sectional and longitudinal research (Gardiner & Iarocci, 2018; Gilotty et al., 2002; Pugliese et al., 2016).

The relationship between EF difficulties and adaptive functioning can be understood through various potential pathways. Given that EF involve the cognitive processes required for executing goal-directed behaviors, impaired EF could lead to difficulties in effectively carrying out goal-directed activities in everyday life. For instance, working memory skills which concern the ability to hold and manipulate information in mind is fundamental for tasks that require following multi-step instructions or solving complex problems (Diamond, 2013). Children with poor working memory skills may present with learning difficulties or as academic underachievers, which can affect their educational attainment and impede their future career opportunities (Gathercole & Packiam Alloway, 2008). Moreover, inhibition skills, which concern the ability to suppress impulsive behaviors and ignore irrelevant stimuli, is essential for carrying out appropriate social behavior and self-control (Diamond, 2013). Children with poor inhibition skills could present with impulsive behaviors and difficulties with adhering to social norms, which could lead to later social difficulties (Gewirtz et al., 2009). Finally, mental flexibility skills concern the ability to adapt one’s thoughts and behaviors to changing circumstances (Diamond, 2013). Children with difficulties in mental flexibility may struggle with problem-solving, decision-making, and adapting to new situations or demands in educational or occupational contexts, and research has shown that poor decision-making may negatively impact adaptive function in individuals with ASD (Gaeth et al., 2016; Hosozawa et al., 2021).

In the context of emerging adulthood, it is evident that early EF difficulties may have an independent impact on adaptive functioning, beyond the ASD diagnosis or co-occurring symptom level (depressive symptoms or total problems) Both ASD symptoms and co-occurring symptoms may fluctuate over time (Hartman et al., 2016; Magiati et al., 2014; McCauley et al., 2020). In contrast, EF have been identified as a persistent difficulty in individuals with ASD, demonstrating its reliability as an endophenotype (Craig et al., 2016; Fossum et al., 2021*; Hartman et al., 2016; Rommelse et al., 2011). The current findings indicate a link between EF difficulties and lower adaptive function across various domains such as family relations, work adjustment, and social functioning. Notably, the prospective predictive value of EF difficulties is not restricted to the domain of adaptive functioning. Previous research has identified a longitudinal association between early EF difficulties and higher levels of internalizing and externalizing psychopathology symptoms in emerging adults with ASD (Fossum et al., 2023*; Hollocks et al., 2022). Taken together, these findings suggest that EF may influence a range of outcomes over time in individuals with ASD and may indicate that research on EF can offer valuable insights into the causes of difficulties, prognosis, and treatment planning in ASD populations.

### Implications

Knowledge about early factors that are linked to later adaptive function is important for clinical and research purposes. The findings of EF difficulties in young age as a predictor of adaptive functioning in emerging adulthood highlight the importance of EF for real-life functioning. This is particularly relevant for individuals with ASD, who have a higher prevalence of EF difficulties. Our findings suggest that addressing EF difficulties during childhood could be crucial for improving adaptive functioning in adulthood. One approach would be targeting EF difficulties by providing support for and compensatory strategies which could improve adaptive function outcomes despite living with cognitive difficulties. Goal Management Training is one relevant cognitive remediation intervention targeting EF difficulties that has demonstrated positive effects on everyday life outcomes in various clinical populations (Haugen et al., 2022; Stamenova & Levine, 2018). Moreover, interventions that improve EF skills directly or contribute to optimal EF development could also possibly improve functional outcome. Early Intensive Behavioral Interventions, potentially supporting EF (Skogli et al., 2020) have reported significant improvements in adaptive behavior in preschool children with ASD (Eldevik et al., 2009; Magiati et al., 2012).

### Strengths and Limitations

Using a longitudinal study allows for investigating long-term adaptive functioning outcomes in individuals with ASD, a population more often studied in childhood/adolescence (Rommelse & Hartman, 2016). Longitudinal studies provide insight on prognosis and the long-term development of individuals diagnosed with ASD in young age (Steinhausen et al., 2016), differing from cross-sectional study samples. Our ASD sample comprised individuals without ID, which reduces variability and complexity introduced by comorbid ID that per definition is associated with substantially impaired adaptive functioning. This can allow for a more precise examination of the specific impacts of ASD diagnosis, co-occurring psychopathology and EF on adaptive functioning. The TD group allows for comparing self-reported functional impairment across groups. A commendable retention rate (75%), the use of multiple informants and data collection methods reduce the risk of bias. Using a self-report measure of depressive symptoms combined with a parent-report measure of psychopathology symptoms is in line with the recommendation of multiple informants (van der Ende et al., 2012), and enhance the robustness of the findings. Moreover, the use of a self-report measure of adaptive functioning illuminates the subjective experiences of a population that is frequently spoken for by others.

A limitation is the lack of research on psychometric properties of WFIRS and SMFQ in ASD populations. A small sample size may reduce statistical power and the limited number of females in the ASD group restricted sex analysis. Group differences in adaptive function subdomains were not controlled for multiple comparisons, to avoid the risk of type II errors due to the small sample size. The high number of covariates and predictors in the regression analyses also increased risk of type II errors, which should be kept in mind when interpreting the non-significant interaction terms.The current study exclusively examined individual factors, suggesting future research could include other predictors such as access to treatment, family and social networks, educational and occupational environments (Kirby et al., 2016).

## Conclusion

The findings of our study suggest that a notable proportion of individuals diagnosed with ASD in childhood or adolescence demonstrated favorable outcomes in emerging adulthood, including achievements in education, employment, and friendship. At group level, the ASD individuals nevertheless reported significantly lower adaptive functioning when compared to their TD peers. Our results indicated that greater EF difficulties during childhood/adolescence predicted lower self-reported adaptive functioning in emerging adulthood, among individuals with and without ASD. The findings imply that interventions targeting EF difficulties could potentially benefit the real-life functioning of individuals with ASD as they transition into adulthood.

## Footnotes

1 In line with the terminology used by the Norwegian Autism Society and the preferences of one of the authors with an autism diagnosis, this paper applies person-first language.

2 Several publications on longitudinal outcomes for individuals with neurodevelopmental disorders are derived from a Norwegian dataset, the LINEUP study, which was also used in the current study. Asterisks (*) are placed next to all citations from the LINEUP study, to maximize transparency.

## References

[CR1] Achenbach, T. M., & Rescorla, L. (2001). *Manual for the ASEBA school-age forms & profiles: An integrated system of multi-informant assessment*. Burlington, VT.

[CR2] Achenbach, T. M., & Rescorla, L. (2003). *Manual for the ASEBA adult forms & profiles*. University of Vermont, Research Center for Children, Youth, & Families.

[CR3] Agresti, A., & Finlay, B. (2009). *Statistical methods for the social sciences*. Pearson Prentice Hall.

[CR4] American Psychiatric Association (2013). *Diagnostic and statistical manual of mental disorders* (5th ed.). 10.1176/appi.books.9780890425596

[CR5] Andersen, P. N., Hovik, K. T., Skogli, E. W., Egeland, J., & Øie, M. (2013). Symptoms of ADHD in children with high-functioning autism are related to impaired verbal working memory and verbal delayed recall. *PloS One*, *8*(5), e64842. 10.1371/journal.pone.0064842 23717667 10.1371/journal.pone.0064842 PMC3661504

[CR6] Andersen, P. N., Orm, S., Fossum, I. N., Øie, M. G., & Skogli, E. W. (2023). Adolescence internalizing problems as a mediator between autism diagnosis in childhood and quality of life in emerging adults with and without autism: A 10-year longitudinal study. *Bmc Psychiatry*, *23*(1), 149. 10.1186/s12888-023-04635-w 36894901 10.1186/s12888-023-04635-w PMC9996871

[CR7] Angold, A., Costello, E. J., Messer, S. C., Pickles, A., Winder, F., & Silver, D. (1995). The development of a short questionnaire for use in epidemiological studies of depression in children and adolescents. *International Journal of Methods in Psychiatric Research*, *5*, 1–12.

[CR8] Arnett, J. J. (2000). Emerging adulthood: A theory of development from the late teens through the twenties. *American Psychologist*, *55*(5), 469. 10.1037/0003-066X.55.5.469 10842426

[CR9] Aydin, Ü., Capp, S. J., Tye, C., Colvert, E., Lau-Zhu, A., Rijsdijk, F., Palmer, J., & McLoughlin, G. (2022). Quality of life, functional impairment and continuous performance task event‐related potentials (ERPs) in young adults with ADHD and autism: A twin study. *JCPP Advances*, *2*(3). 10.1002/jcv2.12090 10.1002/jcv2.12090 PMC10242939 37431386

[CR10] Bagetta, P., & Alexander, P. A. (2016). Conceptualization and operationalization of executive function. *Mind Brain and Education*, *10*(1), 10–33. 10.1111/mbe.12100

[CR11] Canu, W. H., Hartung, C. M., Stevens, A. E., & Lefler, E. K. (2020). Psychometric properties of the Weiss Functional Impairment Rating Scale: Evidence for utility in research, assessment, and treatment of ADHD in emerging adults. *Journal of Attention Disorders*, *24*(12), 1648–1660. 10.1177/1087054716661421 27481918 10.1177/1087054716661421

[CR12] Cederlund, M., Hagberg, B., Billstedt, E., Gillberg, I. C., & Gillberg, C. (2008). Asperger syndrome and autism: A comparative longitudinal follow-up study more than 5 years after original diagnosis. *Journal of Autism and Developmental Disorders*, *38*, 72–85. 10.1007/s10803-007-0364-6 17340200 10.1007/s10803-007-0364-6

[CR13] Chandler, S., Leno, C., White, V., Yorke, P., Hollocks, I., Baird, M. J., & Charman, G., T (2022). Pathways to adaptive functioning in autism from early childhood to adolescence. *Autism Research*, *15*(10), 1883–1893. 35899846 10.1002/aur.2785 PMC9796413

[CR14] Clarke, E. B., McCauley, J. B., & Lord, C. (2021). Post–high school daily living skills in autism spectrum disorder. *Journal of the American Academy of Child & Adolescent Psychiatry*, *60*(8), 978–985. 33220430 10.1016/j.jaac.2020.11.008 PMC8128936

[CR15] Cohen, J. (1992). Statistical power analysis. *Current Directions in Psychological Science Autism*, *1*(3), 98–101.

[CR16] Copeland, W. E., Wolke, D., Shanahan, L., & Costello, E. J. (2015). Adult functional outcomes of common childhood psychiatric problems: A prospective, longitudinal study. *JAMA Psychiatry*, *72*(9), 892–899. 10.1001/jamapsychiatry.2015.0730 26176785 10.1001/jamapsychiatry.2015.0730 PMC4706225

[CR17] Costello, E. J., & Angold, A. (1988). Scales to assess child and adolescent depression: Checklists, screens, and nets. *Journal of the American Academy of Child & Adolescent Psychiatry*, *27*(6), 726–737. 10.1097/00004583-198811000-00011 3058677 10.1097/00004583-198811000-00011

[CR18] Costello, E. J., Benjamin, R., Angold, A., & Silver, D. (1991). Mood variability in adolescents: A study of depressed, nondepressed and comorbid patients. *Journal of Affective Disorders*, *23*(4), 199–212. 10.1016/0165-0327(91)90101-W 1791265 10.1016/0165-0327(91)90101-w

[CR19] Craig, F., Margari, F., Legrottaglie, A. R., Palumbi, R., de Giambattista, C., & Margari, L. (2016). A review of executive function deficits in autism spectrum disorder and attention-deficit/hyperactivity disorder. *Neuropsychiatric Disease and Treatment*, *12*, 1191–1202. 10.2147/NDT.S104620 27274255 10.2147/NDT.S104620 PMC4869784

[CR20] Daniels, A. M., & Mandell, D. S. (2014). Explaining differences in age at autism spectrum disorder diagnosis: A critical review. *Autism*, *18*(5), 583–597. 23787411 10.1177/1362361313480277 PMC4775077

[CR21] Delis, D., Kaplan, E., & Kramer, J. (2001). *Delis-Kaplan executive function system (D-KEFS). Norwegian version*. Pearson Assessment.

[CR22] Demetriou, E. A., Lampit, A., Quintana, D. S., Naismith, S. L., Song, Y. J. C., Pye, J. E., Hickie, I., & Guastella, A. J. (2017). Autism spectrum disorders: A meta-analysis of executive function. *Molecular Psychiatry*, *23*(5), 1198–1204. 10.1038/mp.2017.75 28439105 10.1038/mp.2017.75 PMC5984099

[CR23] Diamond, A. (2013). Executive functions. *Annual Review of Psychology*, *64*, 135–168. 10.1146/annurev-psych-113011-143750 23020641 10.1146/annurev-psych-113011-143750 PMC4084861

[CR24] DuPaul, G. J., Power, T. J., Anastopoulos, A. D., & Reid, R. (1998). *ADHD rating Scale—IV: Checklists, norms, and clinical interpretation*. Guilford Press.

[CR25] Ehlers, S., Gillberg, C., & Wing, L. (1999). A screening questionnaire for Asperger syndrome and other high-functioning autism spectrum disorders in school age children. *Journal of Autism and Developmental Disorders*, *29*(2), 129–141. 10.1023/A:1023040610384 10382133 10.1023/a:1023040610384

[CR26] Eldevik, S., Hastings, R. P., Hughes, J. C., Jahr, E., Eikeseth, S., & Cross, S. (2009). Meta-analysis of early intensive behavioral intervention for children with autism. *Journal of Clinical Child and Adolescent Psychology*, *38*(3), 439–450. 10.1080/15374410902851739 19437303 10.1080/15374410902851739

[CR27] Farley, M. A., McMahon, W. M., Fombonne, E., Jenson, W. R., Miller, J., Gardner, M., Block, H., Pingree, C. B., Ritvo, E. R., Ritvo, R. A., & Coon, H. (2009). Twenty-year outcome for individuals with autism and average or near-average cognitive abilities. *Autism Research*, *2*(2), 109–118. 10.1002/aur.69 19455645 10.1002/aur.69

[CR28] Fjermestad, K. W., Wergeland, G. J., Rogde, A., Bjaastad, J. F., Heiervang, E., & Haugland, B. S. M. (2020). School-based targeted prevention compared to specialist mental health treatment for youth anxiety. *Child and Adolescent Mental Health*, *25*(2), 102–109. 10.1111/camh.12366 32307836 10.1111/camh.12366

[CR37] Forbes, G., Kent, R., Charman, T., Baird, G., Pickles, A., & Simonoff, E. (2023). How do autistic people fare in adult life and can we predict it from childhood? *Autism Research*, *16*(2), 458–473. 36519265 10.1002/aur.2868 PMC10947100

[CR29] Fossum, I. N., Andersen, P. N., Oie, M. G., & Skogli, E. W. (2021). Development of executive functioning from childhood to young adulthood in autism spectrum disorder and attention-deficit/hyperactivity disorder: A 10-year longitudinal study. *Neuropsychology*, *35*(8), 809–821. 10.1037/neu0000768 34570540 10.1037/neu0000768

[CR30] Fossum, I. N., Orm, S., Andersen, P. N., Geurts, H. M., Øie, M. G., & Skogli, E. W. (2023). Childhood executive function predicts internalizing and externalizing symptoms in emerging adults with and without autism: A 10-year longitudinal study. *Developmental Neuropsychology*, *48*(3), 97–111. 10.1080/87565641.2023.2206663 37154789 10.1080/87565641.2023.2206663

[CR31] Gaeth, G. J., Levin, I. P., Jain, G., & Burke, E. V. (2016). Toward understanding everyday decision making by adults across the autism spectrum. *Judgment and Decision Making*, *11*(6), 537–547. 10.1017/S1930297500004757

[CR32] Gardiner, E., & Iarocci, G. (2018). Everyday executive function predicts adaptive and internalizing behavior among children with and without autism spectrum disorder. *Autism Research*, *11*(2), 284–295. 10.1002/aur.1877 28960841 10.1002/aur.1877

[CR33] Gathercole, S., & Packiam Alloway, T. (2008). Working memory and learning: A practical guide for teachers.

[CR34] Gewirtz, S., Stanton-Chapman, T. L., & Reeve, R. E. (2009). Can inhibition at preschool age predict attention‐deficit/hyperactivity disorder symptoms and social difficulties in third grade? *Early Child Development and Care*, *179*(3), 353–368. 10.1080/03004430601119885

[CR35] Gillberg, C., & Fernell, E. (2014). Autism plus versus autism pure. *Journal of Autism and Developmental Disorders*, *44*, 3274–3276. 24958434 10.1007/s10803-014-2163-1

[CR36] Gilotty, L., Kenworthy, L., Sirian, L., Black, D. O., & Wagner, A. E. (2002). Adaptive skills and executive function in autism spectrum disorders. *Child Neuropsychology*, *8*(4), 241–248. 10.1076/chin.8.4.241.13504 12759821 10.1076/chin.8.4.241.13504

[CR38] Hartman, C. A., Geurts, H. M., Franke, B., Buitelaar, J. K., & Rommelse, N. N. (2016). Changing ASD-ADHD symptom co-occurrence across the lifespan with adolescence as crucial time window: Illustrating the need to go beyond childhood. *Neuroscience and Biobehavioral Reviews*, *71*, 529–541. 10.1016/j.neubiorev.2016.09.003 27629802 10.1016/j.neubiorev.2016.09.003

[CR39] Haugan, A. J., Sund, A. M., Thomsen, P. H., Lydersen, S., & Novik, T. S. (2021). Psychometric properties of the Weiss Functional Impairment Rating Scale parent and self-reports in a Norwegian clinical sample of adolescents treated for ADHD. *Nordic Journal of Psychiatry*, *75*(1), 63–72. 10.1080/08039488.2020.1795252 32749193 10.1080/08039488.2020.1795252

[CR40] Helles, A., Gillberg, C. I., Gillberg, C., & Billstedt, E. (2015). Asperger syndrome in males over two decades: Stability and predictors of diagnosis. *Journal of Child Psychology and Psychiatry*, *56*(6), 711–718. 10.1111/jcpp.12334 25283685 10.1111/jcpp.12334

[CR41] Helles, A., Gillberg, I. C., Gillberg, C., & Billstedt, E. (2017). Asperger syndrome in males over two decades: Quality of life in relation to diagnostic stability and psychiatric comorbidity. *Autism*, *21*(4), 458–469. 10.1177/1362361316650090 27233289 10.1177/1362361316650090

[CR42] Hollocks, M. J., Charman, T., Baird, G., Lord, C., Pickles, A., & Simonoff, E. (2022). Exploring the impact of adolescent cognitive inflexibility on emotional and behavioural problems experienced by autistic adults. *Autism*, *26*(5), 1229–1241. 10.1177/13623613211046160 34579554 10.1177/13623613211046160 PMC9340135

[CR43] Hosozawa, M., Mandy, W., Cable, N., & Flouri, E. (2021). The role of decision-making in psychological wellbeing and risky behaviours in autistic adolescents without ADHD: Longitudinal evidence from the UK Millennium cohort study. *Journal of Autism and Developmental Disorders*, *51*(9), 3212–3223. 10.1007/s10803-020-04783-y 33196916 10.1007/s10803-020-04783-y PMC8349343

[CR44] Howlin, P., & Magiati, I. (2017). Autism spectrum disorder: Outcomes in adulthood. *Current Opinion in Psychiatry*, *30*(2), 69–76. 10.1097/YCO.0000000000000308 28067726 10.1097/YCO.0000000000000308

[CR45] Howlin, P., Moss, P., Savage, S., & Rutter, M. (2013). Social outcomes in mid- to later adulthood among individuals diagnosed with autism and average nonverbal IQ as children. *Journal of the American Academy of Child and Adolescent Psychiatry*, *52*(6), 572–581e571. 10.1016/j.jaac.2013.02.017 23702446 10.1016/j.jaac.2013.02.017

[CR49] 10.1007/s10802-018-0493-8

[CR47] Karr, J. E., Areshenkoff, C. N., Rast, P., Hofer, S. M., Iverson, G. L., & Garcia-Barrera, M. A. (2018). The unity and diversity of executive functions: A systematic review and re-analysis of latent variable studies. *Psychological Bulletin*, *144*(11), 1147. 30080055 10.1037/bul0000160 PMC6197939

[CR46] Kaufman, J., Birmaher, B., Brent, D., Rao, U., Flynn, C., Moreci, P., Williamson, D., & Ryan, N. (1997). Schedule for affective disorders and schizophrenia for school-age children-present and lifetime version (K-SADS-PL): Initial reliability and validity data. *Journal of the American Academy of Child and Adolescent Psychiatry*, *36*(7), 980–988. 10.1097/00004583-199707000-00021 9204677 10.1097/00004583-199707000-00021

[CR48] Kenny, L., Cribb, S. J., & Pellicano, E. (2019). Childhood executive function predicts later autistic features and adaptive behavior in young autistic people: A 12-year prospective study. *Journal of Abnormal Child Psychology*, *47*(6), 1089–1099. 30421376 10.1007/s10802-018-0493-8

[CR50] Kirby, A. V., Baranek, G. T., & Fox, L. (2016). Longitudinal predictors of outcomes for adults with autism spectrum disorder: Systematic review. *OTJR: Occupation Participation and Health*, *36*(2), 55–64. 10.1177/1539449216650182 27504878 10.1177/1539449216650182

[CR51] Kornør, H., & Jozefiak, T. (2012). Måleegenskaper ved den norske versjonen av child behavior checklist (CBCL).* PsykTestBarn*, *1*, 3. 10.21337/0014

[CR90] Kraper, C. K., Kenworthy, L., Popal, H., Martin, A., & Wallace, G. L. (2017). The gap between adaptive behavior and intelligence in autism persists into young adulthood and is linked to psychiatric co-morbidities. *Journal of Autism and Developmental Disorders*, *47*, 3007–3017. 10.1007/s10803-017-3213-210.1007/s10803-017-3213-228710532

[CR52] Kuo, E. S., Stoep, A. V., & Stewart, D. G. (2005). Using the short mood and feelings questionnaire to detect depression in detained adolescents. *Assessment*, *12*(4), 374–383. 10.1177/1073191105279984 16244118 10.1177/1073191105279984

[CR53] Lai, M. C., Lombardo, M. V., & Baron-Cohen, S. (2014). Autism. *The Lancet*, *383*(9920), 896–910. 10.1016/s0140-6736(13)61539-1 10.1016/S0140-6736(13)61539-1 24074734

[CR54] Lau-Zhu, A., Fritz, A., & McLoughlin, G. (2019). Overlaps and distinctions between attention deficit/hyperactivity disorder and autism spectrum disorder in young adulthood: Systematic review and guiding framework for EEG-imaging research. *Neuroscience and Biobehavioral Reviews*, *96*, 93–115. 10.1016/j.neubiorev.2018.10.009 30367918 10.1016/j.neubiorev.2018.10.009 PMC6331660

[CR55] Magiati, I., Tay, X. W., & Howlin, P. (2012). Early comprehensive behaviorally based interventions for children with autism spectrum disorders: A summary of findings from recent reviews and meta-analyses. *Neuropsychiatry*, *2*(6), 543. 10.2217/npy.12.59

[CR56] Magiati, I., Tay, X. W., & Howlin, P. (2014). Cognitive, language, social and behavioural outcomes in adults with autism spectrum disorders: A systematic review of longitudinal follow-up studies in adulthood. *Clinical Psychology Review*, *34*(1), 73–86. 10.1016/j.cpr.2013.11.002 24424351 10.1016/j.cpr.2013.11.002

[CR57] Mandell, D. S., Novak, M. M., & Zubritsky, C. D. (2005). Factors associated with age of diagnosis among children with autism spectrum disorders. *Pediatrics*, *116*(6), 1480–1486. 16322174 10.1542/peds.2005-0185 PMC2861294

[CR58] Mason, D., Capp, S. J., Stewart, G. R., Kempton, M. J., Glaser, K., Howlin, P., & Happé, F. (2021). A meta-analysis of outcome studies of autistic adults: Quantifying effect size, quality, and meta-regression. *Journal of Autism and Developmental Disorders*, *51*, 3165–3179. 10.1007/s10803-020-04763-2 33200352 10.1007/s10803-020-04763-2 PMC8349337

[CR59] Mattila, M. L., Hurtig, T., Haapsamo, H., Jussila, K., Kuusikko-Gauffin, S., Kielinen, M., Linna, S. L., Ebeling, H., Bloigu, R., Joskitt, L., Pauls, D. L., & Moilanen, I. (2010). Comorbid psychiatric disorders associated with Asperger syndrome/high-functioning autism: A community- and clinic-based study. *Journal of Autism and Developmental Disorders*, *40*(9), 1080–1093. 10.1007/s10803-010-0958-2 20177765 10.1007/s10803-010-0958-2

[CR91] Mazefsky, C. A., Borue, X., Day, T. N., & Minshew, N. J. (2014). Emotion regulation patterns in adolescents with high‐functioning autism spectrum disorder: Comparison to typically developing adolescents and association with psychiatric symptoms. *Autism Research*, *7*(3), 344–354. 10.1002/aur.1366 10.1002/aur.1366 PMC4136477 24610869

[CR60] McCauley, J. B., Elias, R., & Lord, C. (2020). Trajectories of co-occurring psychopathology symptoms in autism from late childhood to adulthood. *Development and Psychopathology*, *32*(4), 1287–1302. 10.1017/S0954579420000826 32677592 10.1017/S0954579420000826 PMC7655668

[CR61] Micai, M., Fatta, L. M., Gila, L., Caruso, A., Salvitti, T., Fulceri, F., & Scattoni, M. L. (2024). Prevalence of co-occurring conditions in children and adults with autism spectrum disorder: A systematic review and meta-analysis. *Neuroscience & Biobehavioral Reviews*, *155*, 105436. 10.1016/j.neubiorev.2023.105436 37913872

[CR62] Miyake, A., Friedman, N. P., Emerson, M. J., Witzki, A. H., Howerter, A., & Wager, T. D. (2000). The unity and diversity of executive functions and their contributions to complex frontal lobe tasks: A latent variable analysis. *Cognitive Psychology*, *41*(1), 49–100. 10.1006/cogp.1999.0734 10945922 10.1006/cogp.1999.0734

[CR63] Nyrenius, J., & Billstedt, E. (2020). The functional impact of cognition in adults with autism spectrum disorders. *Nordic Journal of Psychiatry*, *74*(3), 220–225. 31762354 10.1080/08039488.2019.1694698

[CR64] O’Hearn, K., Asato, M., Ordaz, S., & Luna, B. (2008). Neurodevelopment and executive function in autism. *Development and Psychopathology*, *20*(04), 1103–1132. 10.1017/S0954579408000527 18838033 10.1017/S0954579408000527

[CR65] Øie, M. G., Sundet, K., Haug, E., Zeiner, P., Klungsøyr, O., & Rund, B. R. (2021). Cognitive performance in early-onset schizophrenia and attention-deficit/hyperactivity disorder: A 25-year follow-up study. *Frontiers in Psychology*, *11*, 606365. 33519613 10.3389/fpsyg.2020.606365 PMC7841368

[CR66] Orm, S., Andersen, P. N., Fossum, I. N., Øie, M. G., & Skogli, E. W. (2022a). Brief report: Autism spectrum disorder diagnostic persistence in a 10-year longitudinal study. *Research in Autism Spectrum Disorders*, *97*, 102007. 10.1016/j.rasd.2022.102007

[CR67] Orm, S., Pollak, Y., Fossum, I. N., Andersen, P. N., Øie, M. G., & Skogli, E. W. (2022b). Decision-making and risky behavior in individuals with attention-deficit/hyperactivity disorder: A 10-year longitudinal study. *Developmental Neuropsychology*, *47*(4), 193–209. 10.1080/87565641.2022.2082430 35642565 10.1080/87565641.2022.2082430

[CR68] Orm, S., Øie, M. G., Fossum, I. N., Fjermestad, K., Andersen, P. N., & Skogli, E. W. (2023). Predictors of quality of life and functional impairments in emerging adults with and without ADHD: A 10-year longitudinal study. *Journal of Attention Disorders*, *27*(5), 458–469. 10.1177/10870547231153962 36779541 10.1177/10870547231153962

[CR92] Pandolfi, V., Magyar, C. I., & Dill, C. A. (2012). An initial psychometric evaluation of the CBCL 6–18 in a sample of youth with autism spectrum disorders. *Research in Autism Spectrum Disorders*, *6*(1), 96–108. 10.1016/j.rasd.2011.03.009 10.1016/j.rasd.2011.03.009 PMC3207215 22059091

[CR69] Pellicano, E. (2012). The development of executive function in autism. *Autism Research and Treatment*, *2012*, 146132. 10.1155/2012/146132 22934168 10.1155/2012/146132 PMC3420556

[CR70] Pellicano, E. (2013). Testing the predictive power of cognitive atypicalities in autistic children: Evidence from a 3-year follow‐up study. *Autism Research*, *6*(4), 258–267. 10.1002/aur.1286 23495146 10.1002/aur.1286

[CR71] Pickles, A., McCauley, J. B., Pepa, L. A., Huerta, M., & Lord, C. (2020). The adult outcome of children referred for autism: Typology and prediction from childhood. *Journal of Child Psychology and Psychiatry*, *61*(7), 760–767. 31957035 10.1111/jcpp.13180 PMC7384105

[CR72] Pugliese, C. E., Anthony, L., Strang, J. F., Dudley, K., Wallace, G. L., & Kenworthy, L. (2015). Increasing adaptive behavior skill deficits from childhood to adolescence in autism spectrum disorder: Role of executive function. *Journal of Autism and Developmental Disorders*, *45*, 1579–1587. 10.1007/s10803-014-2309-1 25398602 10.1007/s10803-014-2309-1 PMC4433442

[CR73] Pugliese, C. E., Anthony, L. G., Strang, J. F., Dudley, K., Wallace, G. L., Naiman, D. Q., & Kenworthy, L. (2016). Longitudinal examination of adaptive behavior in autism spectrum disorders: Influence of executive function. *Journal of Autism and Developmental Disorders*, *46*(2), 467–477. 10.1007/s10803-015-2584-5 26349921 10.1007/s10803-015-2584-5 PMC4726475

[CR74] Romer, A. L., & Pizzagalli, D. A. (2021). Is executive dysfunction a risk marker or consequence of psychopathology? A test of executive function as a prospective predictor and outcome of general psychopathology in the adolescent brain cognitive development study^®^. *Developmental Cognitive Neuroscience*, *51*, 100994. 34332330 10.1016/j.dcn.2021.100994 PMC8340137

[CR76] Rommelse, N. N., & Hartman, C. A. (2016). Review: Changing (shared) heritability of ASD and ADHD across the lifespan. *European Child & Adolescent Psychiatry*, *25*, 213–215. 10.1007/s00787-016-0830-9 26883794 10.1007/s00787-016-0830-9

[CR75] Rommelse, N. N., Geurts, H. M., Franke, B., Buitelaar, J. K., & Hartman, C. A. (2011). A review on cognitive and brain endophenotypes that may be common in autism spectrum disorder and attention-deficit/hyperactivity disorder and facilitate the search for pleiotropic genes. *Neuroscience and Biobehavioral Reviews*, *35*(6), 1363–1396. 10.1016/j.neubiorev.2011.02.015 21382410 10.1016/j.neubiorev.2011.02.015

[CR77] Sheehan, Sheehan, D. V., Lecrubier, Y., Sheehan, K. H., Amorim, P., Janavs, J., Weiller, E., & Dunbar, G. C. (1998). (1998). The Mini-International Neuropsychiatric Interview (MINI): the development and validation of a structured diagnostic psychiatric interview for DSM-IV and ICD-10. *Journal of clinical psychiatry*, *59*(20), 22–33. 9881538

[CR78] Skogli, E. W., Andersen, P. N., & Isaksen, J. (2020). An exploratory study of executive function development in children with autism, after receiving early intensive behavioral training. *Developmental Neurorehabilitation*, *23*(7), 439–447. 10.1080/17518423.2020.1756499 32397778 10.1080/17518423.2020.1756499

[CR79] Stamenova, V., & Levine, B. (2018). Effectiveness of goal management training^®^ in improving executive functions: A meta-analysis. *Neuropsychological Rehabilitation*, *10*, 1569–1599. 10.1080/09602011.2018.1438294 10.1080/09602011.2018.1438294 29540124

[CR80] Statistics Norway (2024). *Completion rates of pupils in upper secondary education.* Retrieved 23.04.24 from Completion rates of pupils in upper secondary education– SSB.

[CR81] Steinhausen, H. C., Jensen, M., C., & Lauritsen, M. B. (2016). A systematic review and meta-analysis of the long-term overall outcome of autism spectrum disorders in adolescence and adulthood. *Acta Psychiatrica Scandinavica*, *133*(6), 445–452. 10.1111/acps.12559 26763353 10.1111/acps.12559

[CR82] Toft, G., Liu, C., Menon, J., Schendel, D., Loss, G., & Ehrenstein, V. (2021). Assessment of educational attainment and employment among individuals with autism spectrum disorder in Denmark. *JAMA Pediatrics*, *175*(6), 601–608. 10.1001/jamapediatrics.2021.0124 33818591 10.1001/jamapediatrics.2021.0124 PMC8022261

[CR83] Turgay, A., Goodman, D. W., Asherson, P., Lasser, R. A., Babcock, T. F., Pucci, M. L., Barkley, R., & Group, A. T. P. M. W (2012). Lifespan persistence of ADHD: The life transition model and its application. *The Journal of Clinical Psychiatry*, *73*(2), 10337. 10.4088/JCP.10m06628 10.4088/JCP.10m06628 22313720

[CR84] van der Ende, J., Verhulst, F. C., & Tiemeier, H. (2012). Agreement of informants on emotional and behavioral problems from childhood to adulthood. *Psychological Assessment*, *24*(2), 293. 21928910 10.1037/a0025500

[CR85] Wechsler, D. (1999). *Wechsler Abbreviated Scale of Intelligence (Norwegian version)*. The Psychological Corporation.

[CR86] Wechsler, D. (2004). *Wechsler Intelligence Scale for Children (Norwegian version)* (4th ed.). ed.). The Psychological Corporation.

[CR87] Weiss, M. D., Wasdell, M. B., & Bomben, M. M. (2005). *Weiss Functional Impairment Rating Scale - Self Report (WFIRS-S)* (Version 2 ed.).

[CR88] Wong, O. W., Barzilay, R., Lam, A. M., Chan, S., Calkins, M. E., Gur, R. E., & Gur, R. C. (2023). Executive function as a generalized determinant of psychopathology and functional outcome in school-aged autism spectrum disorder: A case-control study. *Psychological Medicine*, *53*(10), 4788–4798. 10.1017/s0033291722001787 35912846 10.1017/S0033291722001787 PMC10388326

[CR89] Woolfenden, S., Sarkozy, V., Ridley, G., Coory, M., & Williams, K. (2012). A systematic review of two outcomes in autism spectrum disorder–epilepsy and mortality. *Developmental Medicine & Child Neurology*, *54*(4), 306–312. 10.1111/j.1469-8749.2012.04223.x 22348343 10.1111/j.1469-8749.2012.04223.x

